# Psychological interventions during breast cancer rehabilitation: a randomized controlled trial comparing structured short-term psychotherapy versus non-specific group discussion

**DOI:** 10.1186/s12885-023-11576-w

**Published:** 2023-11-21

**Authors:** David Fauser, Elena Rimalis-Vogt, Johannes Mattes, Matthias Bethge

**Affiliations:** 1 Institute for Social Medicine and Epidemiology, University of Lübeck, Ratzeburger Allee 160, 23562 Lübeck, Germany; 2 VAMED Rehaklinik Ahrenshoop, Dorfstrasse 55, 18347 Ahrenshoop, Germany

**Keywords:** Breast cancer, Psychological interventions, Short-term psychotherapy, Rehabilitation, Randomized controlled trial

## Abstract

**Purpose:**

Psycho-oncological treatment is recommended in cancer rehabilitation as it improves fatigue, anxiety, depression, and quality of life in breast cancer patients. The aim of our study was to compare a structured short-term psychotherapy and a non-specific group discussion provided during breast cancer rehabilitation.

**Methods:**

Breast cancer patients were randomly assigned to structured group short-term psychotherapy or a non-specific group discussion during breast cancer rehabilitation. The patients completed questionnaires at the beginning and end of rehabilitation and three months after rehabilitation. The primary outcome was anxiety. Secondary outcomes were depression, distress, fatigue and health-related quality of life domains.

**Results:**

In total, 160 patients (80 in both groups) were recruited and included in the analysis. There was no significant difference between both groups in the primary outcome anxiety at the end of rehabilitation (difference = -0.2; 95% CI -1.2 to 0.7) and three months after rehabilitation (difference = 0.2; 95% CI -0.9 to 1.3) and in any secondary outcome. Patients in the short-term psychotherapy group with high anxiety levels at baseline reported fewer depressive symptoms at the end of rehabilitation.

**Conclusions:**

Our study showed no difference between structured short-term psychotherapy and a non-specific group discussion. Patients with high baseline anxiety levels were more likely to benefit from short-term structured psychotherapy. Early identification of this subgroup and symptoms of mental illness should occur after initial treatment in breast cancer patients in order to offer a structured treatment for anxiety and depressive symptoms during rehabilitation.

**Trial registration:**

German Clinical Trials Register (DRKS00017571; 08/07/2019).

## Purpose

With approximately 2.3 million new cases in 2020, breast cancer is the leading cause (11.7% of all cancer cases) of global cancer morbidity worldwide [1]. In 2017, 17.7 million disability-adjusted life years (DALY) were caused due to breast cancer, of which 93% were from years of life lost (YLL) and 7% from years of healthy life lost due to disability (YLD) [2]. Because of early screening and better treatment for breast cancer, survival rates have improved steadily over the last few years [3]. In Germany, there are around 69,000 new cases of breast cancer per year, and it is the most common cancer in women in Germany [4]. Breast cancer is a stressful and traumatic event. Especially after acute cancer treatment has been completed, there may be persistent psychological and social problems. This makes it difficult for women to return to their daily routine, often resulting in stress and depression. Individual, interpersonal, and social factors related to cancer can cause multiple psychological distress reactions [5]. The four-week prevalence of mental illness in breast cancer patients in a large German sample was around 42% [6]. Most common were anxiety disorders (16.8%), adjustment disorders (14.4%), and mood disorders (8.7%) [6]. Comparable results have also been reported in an international meta-analysis [7].

One in three cancer patients desires professional psycho-oncological support [8]. Frequently, the needs relate to psychological support, support in coping with everyday life and the disease, and information on the disease [9]. Psycho-oncology services are a crucial element in rehabilitation for breast cancer patients. In Germany, breast cancer patients can receive cancer rehabilitation services after completing primary treatment. Cancer rehabilitation supports coping with the disease and its consequences and – in working-aged women – maintaining or restoring work ability. Cancer rehabilitation programs are mainly provided by the German Pension Insurance. Participation in a rehabilitation program either requires a claim by the person in need or may be initiated directly by the primary cancer treatment facility, i.e. post-acute rehabilitation. Cancer rehabilitation programs are mainly carried out as inpatient programs lasting 3–4 weeks, with a treatment dose of over 60 h [10]. The programs are delivered by an interdisciplinary team and follows evidence-based rehabilitation therapy standards developed by the German Pension Insurance [11]. Rehabilitation therapy standards describe therapy modules and define minimum requirements for delivery (duration and frequency). The rehabilitation therapy standards recommend psychological interventions of at least 90 min per week during rehabilitation [11], as there is strong evidence from systematic reviews and randomized controlled trials [12–17] that psycho-oncological treatment improves fatigue, anxiety, depression, and quality of life in breast cancer patients. These interventions include cognitive behavioral techniques and psycho-educational interventions.

Due to the high diversity of potential psycho-oncological treatments and the lack of comparative studies, the selection of a specific treatment over other treatments is predominantly determined by the preferences of treatment providers in German inpatient rehabilitation. Pragmatic studies could help to incrementally improve real-world rehabilitation practice by analyzing differences between psychological interventions and an active treatment and to identify and to identify effective additional measures for those particularly affected. Therefore, our study aimed to compare a structured short-term psychotherapy and a non-specific group discussion provided in addition to the common psychological treatments recommended by German breast cancer rehabilitation therapy standards.

## Materials and methods

### Study design

To compare structured short-term psychotherapy with a non-specific group discussion during cancer rehabilitation, a randomized controlled trial was conducted. The patients completed questionnaires at the beginning and end of rehabilitation and three months after rehabilitation. The study was run at a German inpatient rehabilitation center in Mecklenburg-Western Pomerania providing rehabilitation for breast cancer survivors. Recruitment took place from March 2019 to January 2020, and participants were followed up until May 2020. The study protocol was approved by the Ethics Committees of the University of Lubeck (19–094) and Greifswald University Medicine (BB 062/19). The trial was registered in the German Clinical Trials Register (DRKS00017571; 08/07/2019). The article was prepared according to the Consolidated Standards of Reporting Trials (CONSORT) [18].

### Participants

Patients aged 29 to 85 years with breast cancer, who had successfully completed initial cancer treatment, were included if they had at least five points on the National Comprehensive Cancer Network’s (NCCN) Distress Thermometer [19, 20] at the beginning of rehabilitation. Patients who were already undergoing psychiatric and/or psychotherapeutic treatment and patients with an initial ductal carcinoma in situ (corresponding to tumor stage 0) were excluded. Patients who met the inclusion criteria were informed about the study, treatment and asked to provide informed consent after their first examination in the rehabilitation center. All patients received a rehabilitation program which included exercise therapy, physiotherapy, basic social counseling, occupational therapy, and psychological seminars and counseling, as well as nutrition counseling.

### Randomization and blinding

Randomization lists were created by the last author using computer-generated random numbers and permuted blocks of four. Allocation concealment was assured by sequentially numbered, sealed, opaque envelopes, and assignment was unknown at the time of recruitment. Blinding of the medical staff and patients was not feasible because of the nature of the study.

### Treatment

#### Structured short-term psychotherapy

Patients in group A received multi-modal rehabilitation and an additional standardized psychotherapeutic group intervention. The structured intervention comprised a maximum of three group sessions, i.e., one session per week, in open groups of a maximum of 12 people, delivered by two oncological physicians, one of whom was also a psychotherapist (third author). The intervention was an additional treatment to the standard rehabilitation treatment. Psycho-oncological treatment modules required by the German Pension Insurance in accordance with rehabilitation therapy standards were unaffected by this study and were fully adhered to by all study participants [11].

Through the intervention, patients could gain first experiences with psychotherapeutic intervention techniques (e.g., mindfulness meditation linked with behavioral therapy measures such as acceptance and self-commitment, as well as specific information on anxiety, fatigue, and motivation). This experience should inform patients as to whether, where, and how the initiated psychotherapeutic process should be continued after rehabilitation. In order to strengthen patients’ self-determination, the intervention should offer help for self-help (e.g., dealing with negative feelings, early recognition of feeling overburdened, learning protective measures). Table 1 describes the structured intervention in line with the Template for Intervention Description and Replication checklist (TIDieR) [21].

**Table 1:** Description of the intervention according to the TIDieR checklist

Brief Name	Structured short-term psychotherapy
*Why*	There is a lack of sufficiently low-threshold and quickly available services for psychotherapeutic care in breast cancer patients. Outpatient psychotherapy is subject to approval and usually requires the consent of an expert. Additionally, patients often avoid admitting anxiety and depressive feelings and refuse to seek professional help. Rehabilitation has a key role in addressing anxiety, depression, worries, fatigue, pessimism and in promoting self-management skills in patients. Short-term psychotherapy during rehabilitation may help to clarify whether, where and how psychotherapy should be continued after rehabilitation.
*What*	The topics of the three group sessions were anxiety, fatigue and motivation. The intervention included learning about and teaching psychotherapeutic behavioral intervention techniques (e.g., mindfulness meditation linked with behavioral therapy measures such as acceptance and self-commitment, as well as specific information on anxiety, fatigue, and motivation). Additionally, the intervention is intended to provide preventive measures to deal with negative feelings and early recognition of exhaustion feeling overburdened. **Session on anxiety**: 1. Instruction for a short moment of silence with “Visualization of the moment”. 2. Educative part with emphasis on the necessity of anxiety and that anxiety is a normal feeling. Explanation based on bottles. One bottle represents the past with the experiences made, one bottle represents future expectations and imagined future. 3. Group discussion on the topic of anxiety. 4. 1–2 physical exercises on the topic of anxiety. 5. Concluding with 5 min of breathing and mindfulness meditation (body scan). **Session on fatigue**: 1. Instruction for a short moment of silence with “Visualization of the moment”. 2. Educational part with explanation of fatigue and differentiation from depression (Questions: What is an acute fatigue and what is a chronic fatigue). Participants were asked how they rate their vitality after starting or after completing cancer treatment compared to the time before the disease. 3. Group discussion on the topic of fatigue. 4. The therapists then gave practical examples of how to deal with fatigue and how to deal with one’s own strength (well-dosed physical activity; regular exercise in combination with breaks). It was explained to the participants that a measurable reduction in fatigue symptoms can be achieved with just a few minutes of intense physical activity. 5. Concluding with 5 min of breathing and mindfulness meditation (body scan). **Session on motivation**: 1. Instruction for a short moment of silence with “Visualization of the moment”. 2. Educational part with emphasis: there is no one clear way, instead many small, complementary motivational impulses to achieve a flexibly adaptable goal. Next steps: Task on the question: “Why motivation?“. Task: Define your own goal (considering that the goal is specific, measurable, appropriate, realistic and timed). 3. Group discussion on the topic of motivation. 4. Explanation of practical help in everyday life, e.g. schedule lazy days and days you don’t have to do anything or meetings for social activities. 5. Concluding with 5 min of breathing and mindfulness meditation (body scan).
*Who provided*	The intervention was delivered by two oncological physicians, one of whom is also a psychotherapist (third author).
*How*	The intervention was conducted face-to-face in open groups of a maximum of 12 people.
*Where*	The intervention was delivered in a German inpatient rehabilitation center in Mecklenburg-Western Pomerania providing rehabilitation services of breast cancer survivors. Group therapy rooms were available for group therapy.
*When and how much*	The intervention comprised a maximum of three sessions, i.e., one session per week. Each session lasted 50 min, which corresponded to a therapy dose of maximum 150 min.
*Tailoring*	Not planned.
*How well*	The number of group sessions was documented. Furthermore, to assess the actual delivered dose in the cancer rehabilitation program, the therapy dose was documented. The rehabilitation teams used the classification of therapeutic interventions developed by the German Pension Insurance to code all treatments. In addition, patients were asked about psycho-oncological contents and learning objectives.

#### Non-specific group discussion

Patients in group B received multi-modal rehabilitation and an additional non-specific open group discussion. This unstructured group discussion comprised a maximum of three group sessions of 50 min each, delivered by two oncological physicians, one of whom was also a psychotherapist (third author). The topics of the sessions were freely chosen by the participants in the first session and included for example anxiety, fatigue, and return to work after rehabilitation. In addition, dealing with rejection, and feelings of guilt and shame were addressed. The topics were freely discussed without using structured behavioral therapy techniques. The individual psycho-oncological therapy offered according to rehabilitation therapy standards was not affected by the study participation [11].

### Outcomes and other measures

#### Primary outcome

The primary outcome of this study was anxiety, because anxiety is one of the most common mental disorders in breast cancer patients [6]. We assessed anxiety with the Hospital Depression and Anxiety Scale (HADS) [22]. The values of this anxiety scale with seven items range from 0 to 21 points. The level of anxiety was assessed at the beginning and the end of the rehabilitation program and 12 weeks after completing the program. The primary endpoint was the 12-week measurement.

#### Secondary outcomes

Secondary outcomes were assessed at the beginning and the end of the rehabilitation program and 12 weeks after completing the program. Depressive symptoms were assessed with the depression module of the HADS [22]. Values of the sum scale range from 0 to 21 points. Normative data of the German general population showed elevated scores (both ≥ 8 points) for the HADS anxiety module in 21.0% and for depression in 23.7% in the total sample [23]. Psychosocial stress was assessed using the NCCN Distress Thermometer [19]. The Distress Thermometer is widely used in Germany [20]. On a thermometer scale of 0 to 10 points, patients can describe how they have felt in the last week (0 = felt very good, 10 = felt very bad). Fatigue was assessed using the Brief Fatigue Inventory (BFI), which measures the severity and impact of fatigue related to cancer and its treatment [24]. The BFI score can take on values from 0 to 10 points. To assess the quality of life we used the 30-item quality of life questionnaire of the European Organization for Research and Treatment of Cancer (EORTC QLQ-C30), which measures role functioning, physical functioning, emotional functioning, social functioning, pain, and global health [25]. Scores on all scales range from 0 to 100 points. Higher scores indicate better health-related quality of life or more severe symptoms and difficulties on the symptom scales. Clinically relevant differences were defined as differences ≥ 10 points [26].

#### Other measures

In addition, we assessed data to describe our study population and the rehabilitation program. At the beginning of the rehabilitation program, we assessed sociodemographic data (age, sex, and employment) and details on the disease and its treatment (staging according to the Union for International Cancer Control, received cytostatic chemotherapy, received or ongoing immunotherapy, ongoing anti-hormonal therapy, polyneuropathy after chemotherapy, and breast conserving surgery or mastectomy), and the level of satisfaction with the result of local tumor treatment (yes, rather yes, rather no, and no). Furthermore, we assessed the type of rehabilitation (post-acute rehabilitation or non-post-acute rehabilitation) and documented treatments during rehabilitation according to the classifications of therapeutic treatments [27]. In addition, in order to check intervention fidelity, patients were asked about psycho-oncological contents (0 to 12 points) and learning objectives (0 to 12 points) at the end of the rehabilitation program with a standardized set of questions.

### Sample size

The minimum clinically important difference on the HADS is between 1 and 2 points [28, 29], with a standard deviation of 3 points. In order to detect a difference of 1.5 points with a t-test, assuming a two-sided error probability of 5% and a power of 80%, an analysis sample of 128 persons would be needed. On the basis of the expected sample attrition because of a 20% nonresponse to the 3-month follow-up questionnaire, we aimed to recruit at least 160 patients.

### Statistical analysis

Descriptive statistics were used to characterize the samples treated in group A and B and to describe the average dose of treatments. We used t-tests or chi^2^ tests to explore baseline group differences.

Patients were analyzed as randomized, i.e. as intended to treat. The outcomes of both groups were compared using linear regression models separately for both follow-up measurement points (end of rehabilitation and three months after rehabilitation). Baseline parameters of the outcomes were considered as covariates in the models. We report adjusted differences between groups with 95% confidence intervals, as well as adjusted predicted estimates (APE) and standard errors (SE). Our handling of missing data used missing at random assumptions. Missing data were imputed with chained equations [30]. Parameters without missing values (age and delivered therapy dose) were included as covariates in the imputation models. We created 20 independent data sets with complete values. We additionally performed a complete case analysis for the primary outcome, i.e. anxiety.

Subgroup analyses were performed to analyze differences between persons with high and low levels of anxiety or depression at baseline (effect modification). We used thresholds of 10 points for the HADS anxiety module and 7 points for the HADS depression module to screen for anxiety or depression at baseline [31]. To test whether the treatment effect was moderated by anxiety or depression at baseline, we included two-way interaction terms in the regression models. We performed post-hoc power analyses to determine the statistical power for the subgroup analysis.

We additionally tested the effect of the pandemic, and generated a variable that indicated if participants had completed their three-month follow-up questionnaire before or after the German Bundestag declared an epidemic situation of national concern due to SARS-CoV-2 by the end of March 2020. From this point on, many different protective measures were implemented in Germany to interrupt chains of infection (e.g., contact and travel restrictions, postponement of scheduled surgeries). This variable was included as an additional main effect in two supplemental models explaining three-month anxiety and depression.

The results of the statistical tests were regarded as significant if the two-sided p-value of a test was less than 0.05. All calculations were performed with Stata SE Version 15.

## Results

### Baseline sample characteristics

**Fig. 1:**
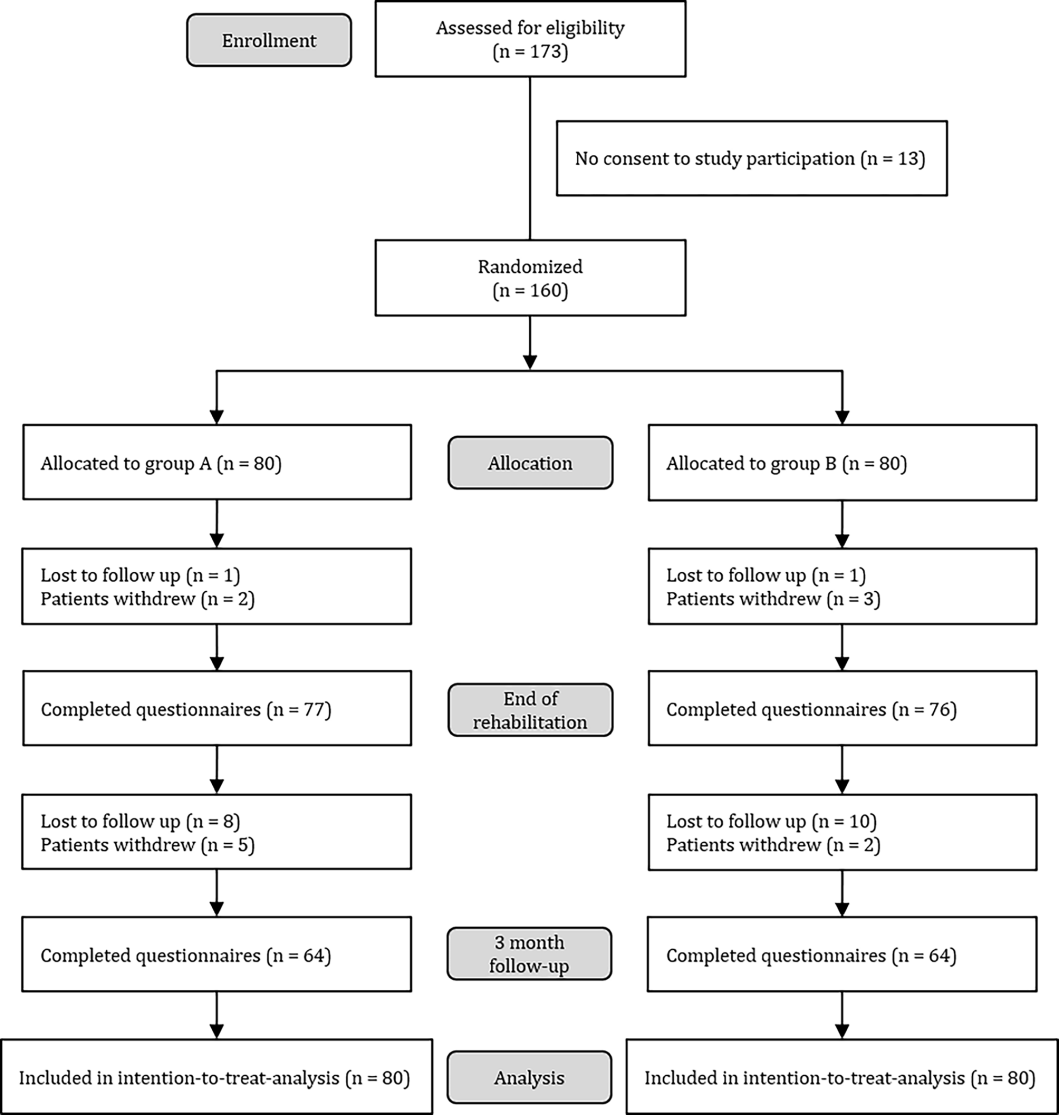
Flow of participants

In total, 173 participants met the inclusion criteria and were informed about the study; 13 women declined to participate in the study. The most common reason was that they did not want to talk about their cancer. In total, 160 participants were treated with short-term psychotherapy (group A: n = 80) or a non-specific group discussion (group B: n = 80). The mean age of the sample was 59.2 years (SD = 10.6). The study flow is shown in Fig. 1. Table 2 presents baseline characteristics by random assignment, identifying no statistically significant differences between group A and B, except in the type of rehabilitation and fatigue. Compared to elevated scores for anxiety and depression in the general population [23], the proportions of elevated scores in our sample were clearly higher, at the start of rehabilitation (anxiety: 70.1%; depression: 51.6%) and at the 3-month follow-up (anxiety: 46.5%; depression: 50%).

**Table 2:** Baseline sample characteristics

	Group A		Group B		p
	n	M (SD) or %	n	M (SD) or %	
***Sociodemographic***					
Age in years (33–85)	80	60.6 (10.7)	80	57.8 (10.4)	0.092
Job situation, %					0.254
Employed: full-time	24	30.0	33	43.4	
Employed: part-time	26	32.5	22	29.0	
Unemployed	1	1.3	2	2.6	
Disability pension or old age pension	29	36.3	19	25.0	
***Cancer disease***					
Time since first diagnosis at start of the rehabilitation in months	78	15.3 (15.4)	74	13.4 (14.6)	0.458
UICC, %					0.219
I	29	36.2	32	40.0	
II	47	58.8	42	52.5	
III	2	2.5	6	7.5	
IV	2	2.5	0	0.0	
***Cancer treatment***					
Cytostatic chemotherapy, %					0.634
Yes	45	56.3	42	52.5	
No	35	43.7	38	47.5	
Immunotherapy, %					1.000
Yes	11	13.8	11	13.8	
No	69	86.2	69	86.2	
Anti-hormonal therapy, %					0.558
Yes	65	81.3	62	77.5	
No	15	18.7	18	22.5	
Polyneuropathy due the therapy, %					0.874
Yes	36	45.0	35	43.8	
No	44	55.0	45	56.2	
Surgery, %					0.291
Breast conserving	69	86.3	64	80.0	
Mastectomy	11	13.7	16	20.0	
Satisfaction with cosmetic outcome, %					0.332
Yes	33	41.8	32	41.0	
Rather yes	36	45.6	29	37.2	
Rather no	4	5.0	10	12.8	
No	6	7.6	7	9.0	
***Rehabilitation type***, **%**					**0.043**
Post-acute rehabilitation	55	68.8	66	82.5	
Non-post-acute rehabilitation	25	31.2	14	17.5	
***Outcomes***					
*HADS*					
Anxiety (0–21)	78	8.8 (3.7)	79	9.5 (4.2)	0.312
Depression (0–21)	78	6.9 (3.5)	79	7.8 (4.1)	0.112
*NCCN Distress Thermometer*					
Distress (0–10)	80	6.1 (1.1)	80	6.2 (1.2)	0.511
*Brief Fatigue Inventory*					
Fatigue (0–10)	80	4.3 (1.4)	80	4.8 (1.5)	**0.038**
*EORTC QLQ-C30*					
Global health (0-100)	78	50.7 (16.1)	80	48.0 (16.9)	0.301
Physical functioning (0-100)	78	68.6 (18.0)	80	64.3 (19.0)	0.139
Role functioning (0-100)	78	48.9 (24.4)	80	46.0 (27.5)	0.486
Emotional functioning (0-100)	78	46.7 (24.1)	80	42.2 (27.4)	0.275
Cognitive functioning (0-100)	78	60.0 (27.6)	80	56.5 (27.6)	0.416
Social functioning (0-100)	78	57.5 (27.1)	80	54.2 (31.1)	0.477
Fatigue (0-100)	78	61.3 (21.2)	80	64.4 (24.7)	0.385
Pain (0-100)	79	46.4 (27.3)	80	52.3 (29.6)	0.195

Deviations in the number of cases in the rows are caused by missing values. Abbreviations: Group A = structured short-term psychotherapy, Group B = non-specific group discussion, SD = standard deviation, UICC = Union for International Cancer Control, HADS = Hospital Depression and Anxiety Scale, NCCN = National Comprehensive Cancer Network, EORTC = European Organization for Research and Treatment of Cancer

### Delivered and received dose

The overall therapy dose (group A: mean = 55.3 h, SD = 16.5; group B: mean = 54.1 h, SD = 19.8; p = 0.664), duration of rehabilitation (group A: mean = 22.2 days, SD = 4.4; group B: mean = 23.0 days, SD = 3.6; p = 0.219), and group sessions provided (group A: mean = 2.8, SD = 0.5; group B: mean = 2.8, SD = 0.5; p = 0.645) were comparable in both groups. In Group A, 5, 6, and 69 and in Group B, 4, 5, and 71 participants received 1, 2, or 3 group sessions, respectively.

Furthermore, there were no statistically significant differences between the groups in terms of patients’ perceived psycho-oncological contents (group A: mean = 7.7, SE = 0.4; group B: mean = 7.9, SE = 0.4; p = 0.747) and learning objectives (group A: mean = 9.0, SE = 0.4; group B: mean = 8.8, SE = 0.4; p = 0.629) through the intervention.

### Outcomes

Table 3 shows the primary and secondary outcomes at the end of rehabilitation and the three-month follow-up. There was no significant difference between groups A and B in the primary outcome (HADS anxiety) at the end of rehabilitation (difference = -0.2; 95% CI -1.2 to 0.7; p = 0.618) and three months after rehabilitation (difference = 0.2; 95% CI -0.9 to 1.3; p = 0.748). The secondary outcomes were also not in favor of group A (Table 3). In an additional complete case analysis, we observed comparable results in the primary outcome at the end (difference = -0.2; 95% CI -1.1 to 0.7; p = 0.650; n = 151) and three months after rehabilitation (difference = 0.5; 95% CI -0.6 to 1.6; p = 0.415; n = 126).

**Table 3:** Primary and secondary outcomes

Outcomes	Adjusted predicted estimates (SE)		Difference	95% CI	p
	Group A	Group B			
***HADS***					
Anxiety					
Beginning of rehabilitation End of rehabilitation 3 months after rehabilitation	8.9 (0.4) 7.3 (0.3) 7.7 (0.4)	9.5 (0.5) 7.6 (0.3) 7.5 (0.4)	-0.2 0.2	-1.2; 0.7 -0.9; 1.3	0.618 0.748
Depression					
Beginning of rehabilitation End of rehabilitation 3 months after rehabilitation	6.9 (0.4) 5.9 (0.3) 6.5 (0.4)	7.8 (0.5) 6.1 (0.3) 6.1 (0.4)	-0.2 0.4	-1.1; 0.6 -0.7; 1.5	0.567 0.480
***NCCN Distress Thermometer***					
Distress					
Beginning of rehabilitation End of rehabilitation 3 months after rehabilitation	6.1 (0.1) 3.3 (0.2) 4.7 (0.3)	6.2 (0.1) 3.6 (0.2) 4.4 (0.3)	-0.3 0.3	-0.9; 0.2 -0.4; 1.0	0.228 0.416
***Brief Fatigue Inventory***					
Fatigue					
Beginning of rehabilitation End of rehabilitation 3 months after rehabilitation	4.3 (0.2) 3.5 (0.1) 4.3 (0.2)	4.8 (0.1) 3.7 (0.1) 3.9 (0.2)	-0.2 0.4	-0.6; 0.2 -0.1; 1.0	0.388 0.118
***EORTC QLQ-C30***					
Global health					
Beginning of rehabilitation End of rehabilitation 3 months after rehabilitation	50.6 (1.8) 60.4 (2.0) 55.7 (2.3)	48.0 (1.9) 62.0 (2.0) 61.0 (2.3)	-1.6 -5.4	-7.1; 4.0 -11.6; 0.8	0.574 0.090
Physical functioning					
Beginning of rehabilitation End of rehabilitation 3 months after rehabilitation	68.6 (2.0) 72.6 (1.8) 70.5 (1.9)	64.3 (2.1) 69.4 (1.8) 73.3 (1.9)	3.2 -2.9	-0.6; 7.0 -8.0; 2.2	0.102 0.270
Role functioning					
Beginning of rehabilitation End of rehabilitation 3 months after rehabilitation	48.7 (2.8) 64.3 (2.5) 56.3 (2.8)	46.0 (3.1) 60.4 (2.5) 61.0 (2.7)	3.9 -4.8	-3.2; 11.0 -12.2; 2.9	0.280 0.224
Emotional functioning					
Beginning of rehabilitation End of rehabilitation 3 months after rehabilitation	46.5 (2.7) 62.4 (2.5) 52.2 (2.6)	42.2 (3.1) 64.6 (2.6) 58.8 (2.6)	-2.3 -6.6	-9.4; 4.9 -14.0; 0.8	0.532 0.079
Cognitive functioning					
Beginning of rehabilitation End of rehabilitation 3 months after rehabilitation	59.8 (3.1) 62.4 (2.0) 64.5 (2.5)	56.5 (3.1) 63.2 (2.0) 69.7 (2.5)	-0.9 -5.2	-6.4; 4.7 -12.5; 2.8	0.761 0.165
Social functioning					
Beginning of rehabilitation End of rehabilitation 3 months after rehabilitation	57.3 (3.1) 65.7 (2.6) 61.1 (3.1)	54.2 (3.5) 68.8 (2.6) 65.9 (3.2)	-3.0 -4.7	-10.2; 4.2 -13.3; 3.8	0.406 0.273
Fatigue					
Beginning of rehabilitation End of rehabilitation 3 months after rehabilitation	61.4 (2.4) 49.4 (2.3) 50.4 (2.7)	64.4 (2.8) 49.3 (2.2) 47.5 (2.7)	0.2 2.9	-6.2; 6.5 -4.6; 10.4	0.960 0.448
Pain					
Beginning of rehabilitation End of rehabilitation 3 months after rehabilitation	46.6 (3.1) 41.8 (1.5) 44.8 (3.0)	52.3 (3.3) 45.0 (1.5) 42.6 (2.9)	-3.2 2.3	-7.4; 1.0 -5.7; 10.3	0.133 0.576

Analyses at the end of rehabilitation and at 3-month follow-up were adjusted for baseline scores. Abbreviations: Group A = structured short-term psychotherapy, Group B = non-specific group discussion, SE = standard error, HADS = Hospital Depression and Anxiety Scale, NCCN = National Comprehensive Cancer Network, EORTC = European Organization for Research and Treatment of Cancer

### Subgroup analysis

We performed subgroup analyses with persons with high and low baseline levels of anxiety or depression. The sample size of the subgroup analysis (high baseline anxiety levels: n = 77) is sufficient to detect the minimum clinically important difference of 1.5 points on the HADS (baseline standard deviation of 3 points) with a t-test, assuming a two-sided error probability of 5% and to achieve a power of 58%.

At the end of rehabilitation, patients in group A with high baseline levels of anxiety had 2 points less on the depression scale compared to patients in group B with high baseline levels of anxiety (HADS depression: difference = -1.9; 95% CI -3.5 to -0.3; p = 0.019) (Table 4). The interaction of treatment and group indicator was statistically significant (p = 0.046). All other subgroup analyses related to baseline anxiety or depression did not reveal statistically significant effect modification.

**Table 4:** Anxiety and depression in subgroups of different levels of anxiety or depression at baseline

Outcomes	Subgroup at baseline	Adjusted predicted estimates (SE)				Difference	95% CI	p
		n	Group A	n	Group B			
***HADS***								
Anxiety								
End of rehabilitation	High anxiety (≥ 10) Low anxiety (< 10)	36 44	9.1 (0.5) 5.5 (0.5)	41 39	10.1 (0.5) 5.3 (0.5)	-1.0 0.2	-2.4; 0.5 -1.2; 1.6	0.183 0.782
3 months after rehabilitation	High anxiety (≥ 10) Low anxiety (< 10)	36 44	8.9 (0.7) 6.4 (0.5)	41 39	9.3 (0.6) 6.0 (0.6)	-0.4 0.5	-2.1; 1.4 -1.2; 2.1	0.670 0.577
End of rehabilitation	High depression (≥ 7) Low depression (< 7)	43 37	8.6 (0.5) 5.4 (0.5)	49 31	9.6 (0.5) 4.8 (0.6)	-1.0 0.5	-2.3; 0.4 -1.1; 2.1	0.161 0.513
3 months after rehabilitation	High depression (≥ 7) Low depression (< 7)	43 37	8.8 (0.6) 6.1 (0.6)	49 31	9.2 (0.5) 5.2 (0.6)	-0.4 0.9	-2.0; 1.2 -0.8; 2.6	0.590 0.300
Depression								
End of rehabilitation	High anxiety (≥ 10) Low anxiety (< 10)	36 44	6.4 (0.6) 4.9 (0.5)	41 39	8.3 (0.6) 4.5 (0.6)	-1.9 0.3	-3.5; -0.3 -1.2; 1.9	**0.019** 0.671
3 months after rehabilitation	High anxiety (≥ 10) Low anxiety (< 10)	36 44	6.8 (0.7) 5.7 (0.6)	41 39	7.8 (0.6) 4.8 (0.6)	-1.0 0.9	-2.8; 0.9 -0.7; 2.6	0.306 0.266
End of rehabilitation	High depression (≥ 7) Low depression (< 7)	43 37	7.3 (0.5) 3.5 (0.5)	49 31	8.5 (0.4) 3.2 (0.6)	-1.2 0.3	-2.5; 0.1 -1.2; 1.8	0.059 0.689
3 months after rehabilitation	High depression (≥ 7) Low depression (< 7)	43 37	7.9 (0.6) 4.3 (0.6)	49 31	7.9 (0.5) 3.8 (0.6)	0.0 0.5	-1.6; 1.5 -1.2; 2.2	0.998 0.590

Abbreviations: Group A = structured short-term psychotherapy, Group B = non-specific group discussion, SE = standard error, HADS = Hospital Depression and Anxiety Scale

In total, 53 participants (IG: n = 26; CG: n = 27) completed their questionnaire at the three-month follow-up after the German Bundestag declared an epidemic situation of national concern due to SARS-CoV-2 by the end of March 2020. In two supplemental models explaining three-month anxiety and depression, we found no evidence of relevant higher impairment in anxiety or depression for patients completing their three-month follow-up questionnaires after the pandemic started to affect Germany.

## Discussion

Breast cancer patients with a moderate stress level were randomly assigned to a structured short-term psychotherapy (group A) or non-specific group discussion (group B) during breast cancer rehabilitation in Germany. Our study showed no difference between structured short-term psychotherapy and non-specific group discussions with respect to anxiety as the primary outcome and all other secondary outcomes in the primary analysis. However, our subgroup analysis showed signs, that participants in group A with high anxiety levels at baseline reported fewer depressive symptoms at the end of rehabilitation.

Our study did not show any evidence of superiority of one of the two psychological interventions and is in accordance with a French randomized trial that evaluated the effects of a structured specific psychosocial intervention that included educational, cognitive behavioral therapy elements and reinforcement in eight sessions over a period of four weeks [32]. Breast cancer patients were randomly assigned to the structured intervention or a support group in which patients could talk about illness and treatment. Cousson-Gélie et al. [32] did not show different outcomes for anxiety and depression for one of the two intervention strategies. Evidence from two meta-analyses of 13 [14] and 27 randomized controlled trials [33] indicates that psychological interventions are effective in reducing anxiety and depression symptoms in breast cancer patients compared with treatment as usual, standard care, or wait-list controls. However, across studies mixed techniques were tested, and there is no evidence that favors one technique over another though effects on anxiety were mainly attributed to long-term psychoeducation of more than eight weeks duration [33].

The result of the subgroup analysis, in that breast cancer patients with high anxiety scores at baseline receiving structured short-term psychotherapy reported lower levels of depression than similar patients receiving unstructured non-specific group discussion, may also indicate that additional and structured psycho-oncological treatment in addition to psycho-oncological treatment in standard rehabilitation care may have added value in high-risk patients, only. This is in line with a German randomized controlled trial that showed that a short-term psycho-oncological intervention led to significant improvements in anxiety and depression only in a high risk group with high levels of anxiety and depression [34]. A further randomized controlled trial analyzed the efficacy of psychodynamic psychotherapy in breast cancer patients with depression (HADS depression score ≥ 8 and depressive disorder diagnosed by the Structured Clinical Interview for Diagnostic and Statistical Manual of Mental Disorders, DSM-IV) [35, 36]. Beutel et al. [36] showed that a psychodynamic psychotherapy led to an improvement of depression and anxiety compared to treatment as usual (written information on cancer counseling centers).

The results of the current study must be interpreted in light of the following limitations. First, the third author had a dual role in that he was the study initiator and, at the same time, a psychotherapist alongside two trained physician psycho-oncologists who delivered the group intervention in groups A and B. This and the single-center setting limit generalizability of our results. Second, participants in both groups (group A and B) took part in weekly therapy sessions. Thus, there is no comparison with participants without an additional group intervention. A three-armed study with a second control group, which would have received only the common dose of psychological treatments specified in the German rehabilitation therapy standards, could have informed whether an additional short-term group has an additional benefit at all. Moreover, the overall treatment dose of 150 min of the short-term psychotherapy might not be sufficient to adequately describe and learn psychotherapeutic behavioral intervention techniques. Third, it was not feasible to blind patients and medical staff. Despite the highly standardized setting and the comparison with an active control group, it cannot be excluded that the patient-reported outcomes were influenced by patient and/or caregiver knowledge about the treatment. Fourth, generalization to other welfare systems and patient groups may be limited because of the intensive and highly standardized rehabilitation programs available for women with breast cancer in Germany. Fifth, no data were collected on how many patients were recommended psychotherapy in group A. This data could have generated important insights analyzing the aim of the structured short-term psychotherapy. Moreover, we did not assess further cancer-specific data on fear of progression and recurrence [37]. Sixth, a few patients who initially did not want to participate in the study agreed to participate at the end of the informed consent interview after they were informed and advised that they would not be actively asked to say anything during the group session although active participation is an important requirement for effective psychotherapy. This might have diluted the effects in both treatment conditions.

Despite these limitations, our study had some strengths. First, the internal validity of the study was assured by its randomized design, which prevented selection bias and yielded comparable study groups. Second, the risk of performance bias was low due the standardized description of the structured psychotherapy, the same dose of treatment in group A and B, and the standardized performance of cancer rehabilitation in German in line with rehabilitation therapy standards.

## Conclusion

Inpatient cancer rehabilitation supports breast cancer patients in accepting this critical life event by providing information, support, and activation of their own resources. In the primary analysis our study did not show an advantage of structured short-term psychotherapy over a non-specific group discussion. The results of the subgroup analysis at the end of rehabilitation suggest that structured short-term psychotherapy may be more helpful compared with a nonspecific approach in breast cancer patients with high risks of psychological comorbidity. Further studies should verify this result, considering adapted inclusion criteria (i.e. high levels of anxiety and depression).

Regardless of our findings, an early identification of symptoms of mental illness and structured treatment of anxiety and depressive symptoms should be provided immediately after extensive multimodality tumor therapy in breast cancer patients. Before establishing new psycho-oncological therapies in cancer rehabilitation or deciding between different psycho-oncological therapies, pragmatic randomized controlled trials should be conducted to assess the added benefit of new components that complement conventional cancer rehabilitation programs.

## Data Availability

The datasets of the current study are available from the corresponding author upon reasonable request.
